# Reduced‐Port Versus Multi‐Port Laparoscopic Colectomy for Colorectal Cancer: A Systematic Review, Meta‐Analysis, and Meta‐Regression

**DOI:** 10.1002/ags3.70264

**Published:** 2026-08-18

**Authors:** Ayesha Shaukat, Saad Ashraf, Hamza Irfan, Muhammad Shahmeer Ullah Shah, Yumna Khabir, Santosh Sah, Um E. Abiha Batool, Muhammad Zain Ul Haq, Omer Bin Khalid Jamil, Aymar Akilimali

**Affiliations:** ^1^ Department of Medicine Dow University of Health Sciences Karachi Pakistan; ^2^ Department of Medicine Shaikh Khalifa Bin Zayed Al Nahyan Medical and Dental College Lahore Pakistan; ^3^ Department of Surgery Dow University of Health Sciences Karachi Pakistan; ^4^ Medical Research Circle Goma Democratic Republic of the Congo

**Keywords:** colorectal cancer, laparoscopic colectomy, meta‐analysis, multi port, reduced port

## Abstract

**Background:**

Reduced‐port laparoscopic colectomy (RPLC) has emerged as an alternative to multi‐port laparoscopic colectomy (MPLC) for colorectal cancer surgery, aiming to reduce operative morbidity and improve cosmetic outcomes. This systematic review and meta‐analysis evaluated and compared operative, postoperative, and oncologic outcomes between RPLC and MPLC.

**Methods:**

Following PRISMA guidelines, we systematically searched PubMed, Cochrane Library, ScienceDirect, and Google Scholar from inception to June 2025. The protocol was registered on PROSPERO (CRD420251078328). Included studies were randomized controlled trials (RCTs) or cohort studies comparing RPLC and MPLC. Primary outcomes were estimated blood loss, operative time, hospital stay, lymph node yield, and postoperative recovery indicators. Secondary outcomes included conversion rates, complications, and Clavien‐Dindo graded adverse events. Risk of bias was evaluated using the Newcastle‐Ottawa Scale and Cochrane RoB 2.0 tool. A random‐effects meta‐regression was performed to explore the impact of high‐stage tumors on the outcomes.

**Results:**

Thirteen studies (*n* = 2897) were included. RPLC significantly reduced operative time (MD: −19.98 min; 95% CI: −29.73 to −10.24, *p* < 0.0001) and estimated blood loss (MD: −13.70 mL; 95% CI: −25.63 to −1.77, *p* = 0.02) without compromising hospital stay, lymph node yield, or complication rates. Meta‐regression revealed tumor stage significantly influenced operative time (*p* = 0.0013).

**Conclusions:**

RPLC appears to provide comparable postoperative outcomes to MPLC with modest intraoperative advantages. As the evidence is largely retrospective and heterogeneous, these findings indicate the feasibility of RPLC rather than its superiority over MPLC. High‐quality clinical trials with longer follow‐up are required to confirm these findings and optimize clinical decision‐making.

**Trial Registration:**

PROSPERO registration ID: CRD420251078328

## Graphical abstract

Reduced‐port laparoscopic colectomy (RPLC) for colorectal cancer offers modest intraoperative benefits, including shorter operative time and lower blood loss, while maintaining comparable postoperative recovery, lymph node yield, and complication rates to multi‐port laparoscopic colectomy (MPLC). Based on 13 studies involving 2897 patients, RPLC appears to be a safe and feasible alternative to MPLC, although current evidence does not establish its superiority and further high‐quality prospective trials are warranted.

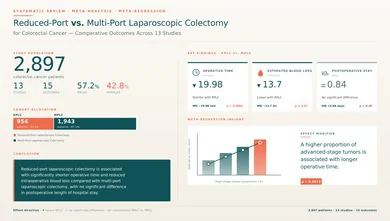

**Abbreviations**
BMI: body mass index
CI: confidence interval
CRC: colorectal cancer
GRADE: Grading of Recommendations Assessment, Development and Evaluation
LFK: Luis Furuya‐Kanamori Index
LMICs: low‐ and middle‐income countries
MD: mean difference
MPLC: multi‐port laparoscopic colectomy
NOS: Newcastle‐Ottawa Scale
PRISMA: Preferred Reporting Items for Systematic Reviews and Meta‐Analyses
PROSPERO: International Prospective Register of Systematic Reviews
RCT: randomized controlled trial
REML: restricted maximum likelihood
RoB 2: Risk of Bias 2.0 tool
RPLC: reduced‐port laparoscopic colectomy
RR: risk ratio
SIL: single‐incision laparoscopy
SILC: single‐incision laparoscopic colectomy

## 1 Introduction

Colorectal cancer or carcinoma (CRC) ranks as the third most frequently diagnosed cancer and stands as the second leading cause of cancer‐related mortality globally [1]. In recent years, significant progress in surgical approaches, anatomical knowledge, and instrumentation has transformed the management of CRC. Laparoscopic colectomy, in particular, has become widely adopted in managing various stages of colorectal malignancies, from early lesions to more advanced cases. Numerous clinical trials have demonstrated that this minimally invasive approach offers several benefits over traditional open surgery, including reduced postoperative pain, a shorter hospital stay, and quicker restoration of bowel function while delivering comparable oncologic outcomes [2, 3].

Laparoscopic colectomy techniques are generally categorized into conventional multi‐port and reduced‐port approaches [4, 5]. Conventional or multi‐port laparoscopic colectomy (MPLC) typically employs five ports: a 12 mm camera port placed supraumbilical and four additional working ports distributed across the abdominal quadrants. This configuration provides optimal instrument triangulation, facilitates dynamic retraction, and allows for active assistance during complex maneuvers [4, 5]. In contrast, the reduced‐port laparoscopic colectomy (RPLC) limits the number of access points, most frequently to two or three ports, depending on the surgical protocol [4, 5]. In the commonly practiced three‐port RPLC, a single 12 mm umbilical camera port and two 5 mm working ports are used, usually located at the left midclavicular line and the suprapubic region. Often performed by a solo surgeon assisted only by a scopist, the RPLC approach aims to reduce postoperative pain, minimize scarring, and improve cosmetic outcomes while maintaining oncological and surgical efficacy [4, 5].

RPLC has been proposed as a safe and effective alternative to MPLC for the treatment of CRC. Studies report shorter operative times, reduced blood loss, similar or higher lymph node yields, and no differences in resection margins or postoperative complication rates. Recovery and cosmetic outcomes may also be improved with the three‐port approach [4, 6, 7]. While the benefits of reduced‐port techniques are well‐documented in other procedures, such as splenectomy, cholecystectomy, and gynecologic surgeries, their application in CRC remains relatively underreported [8].

Currently, no meta‐analyses or systematic reviews specifically compare the efficacy and safety of RPLC with those of MPLC in CRC resection. To address this gap, we conducted a meta‐analysis to evaluate and compare the operative, postoperative, and oncologic outcomes of these two approaches in the management of CRC.

## 2 Methodology

### 2.1 Protocol Registration

This systematic review and meta‐analysis was conducted in alignment with the Preferred Reporting Items for Systematic Reviews and Meta‐Analyses (PRISMA) guidelines [9]. The study protocol was officially recorded in the International Prospective Register of Systematic Reviews (PROSPERO) in 2025, with the registration ID CRD420251078328.

### 2.2 Data Sources and Search Strategy

An extensive and systematic literature search was conducted to identify studies that fulfilled the predefined eligibility criteria from inception till June 2025. The reference lists of all included studies were meticulously reviewed for additional information. The search terms included a combination of PubMed MeSH terms and related keywords, including “hemicolectomy,” “colectomy,” “colorectal cancer OR CRC,” “laparoscopic colectomy,” “reduced‐port,” “three‐port,” “two‐port,” and “multi‐port.” Detailed search strategies specific to each database are provided in Table S1.

### 2.3 Study Selection and Eligibility Criteria

All studies identified through the systematic search were imported into EndNote reference software, version X8.1 (Clarivate Analytics), and duplicates were eliminated. Two reviewers independently screened and selected the studies, with any conflicts resolved through consultation with a third reviewer. Articles that satisfied the initial screening criteria were subjected to full‐text evaluation to verify their eligibility based on the inclusion criteria.

The inclusion criteria for the selected studies were: (i) studies involving adult patients (aged ≥ 18 years) diagnosed with CRC, (ii) studies comparing RPLC using a three‐port or two‐port technique with conventional MPLC using four or five ports, (iii) studies reporting at least one relevant outcome of interest, and (iv) randomized controlled trials (RCTs) or cohort studies. The exclusion criteria were as follows: (i) studies involving patients not diagnosed with CRC, (ii) studies lacking comparative data between RPLC and MPLC, (iii) studies using single‐incision laparoscopy (SIL), robotic‐assisted surgery, or open surgery in either group, (iv) publications not in English, and (v) case reports, case series, editorials, commentaries, letters, conference abstracts, opinion articles, meta‐analyses, and other non‐peer‐reviewed works.

### 2.4 Data Extraction

Two authors independently evaluated the data and Supporting Information, with any discrepancies resolved through consultation with a third author. The following data were extracted for this quantitative synthesis: (i) baseline clinical characteristics of the study populations; (ii) primary outcomes, including estimated blood loss (mL), operative time (minutes), postoperative hospital stay (days), harvested lymph nodes yield (n), time to first flatus (days), and time to first liquid diet (days); and (iii) secondary outcomes, including conversion rate to open surgery, incidence of anastomotic bleeding, postoperative ileus, postoperative complication rates (Clavien‐Dindo Grades 0, 1, 2, 3, and 4), and wound infection.

### 2.5 Quality Assessment

Two independent reviewers conducted the risk of bias and quality assessment, with a third investigator invited to resolve any discrepancies. The Newcastle‐Ottawa Scale (NOS) was used to evaluate the quality of cohorts [10]. Each study received a score out of nine points, with scores of ≥ 7 indicating a low risk of bias, scores between 4 and 6 indicating a moderate risk, and scores of ≤ 3 indicating a high risk of bias. The risk of bias for the RCTs was assessed using the Cochrane Risk of Bias 2.0 (RoB 2) tool [11]. The quality of evidence in this systematic review was evaluated using the Grading of Recommendations Assessment, Development, and Evaluation (GRADE) framework via the GRADEpro GDT software (Table S2) [12].

### 2.6 Statistical Analysis

Two authors conducted the statistical analysis using Review Manager software (RevMan version 5.4; Copenhagen: The Nordic Cochrane Center, The Cochrane Collaboration, 2020). Risk ratios (RRs) were calculated for dichotomous outcomes, while mean differences (MDs) were computed for continuous outcomes. All results were reported with 95% confidence intervals (CIs), and a *p*‐value of ≤ 0.05 was considered statistically significant. Statistical heterogeneity was assessed using the *I* ^2^ statistic, with thresholds of < 20%, 25%–50%, and > 50% indicating low, moderate, and high heterogeneity, respectively. Sensitivity analysis used a leave‐one‐out approach to pinpoint studies contributing to significant heterogeneity. A random‐effects meta‐regression was performed using the restricted maximum likelihood (REML) method to explore whether the proportion of high‐stage tumors (i.e., Stage III or IV, involving lymph nodes or distant metastasis) influenced the effect estimates across outcomes. The MDs served as the dependent variable, and the high‐stage tumors (%) in the RPLC group was included as a continuous moderator. For datasets with fewer than 10 studies, publication bias was evaluated using a DOI plot and the Luis Furuya‐Kanamori (LFK) index, implemented in R version 4.5.1. For 10 or more studies, Egger's test for funnel plot asymmetry was performed to evaluate publication bias.

## 3 Results

### 3.1 Literature Search and Studies Characteristics

A total of 1594 records were retrieved through database searches (PubMed, Cochrane Library, ScienceDirect, and Google Scholar). After removing 628 duplicates, 966 articles remained for initial screening based on titles and abstracts. After this, 92 studies were selected for full‐text assessment to determine eligibility. Following a thorough evaluation, 13 studies met the inclusion criteria and were included in this systematic review and meta‐analysis [4, 5, 6, 7, 8, 13, 14, 15, 16, 17, 18, 19, 20]. A detailed overview of the selection and exclusion process is illustrated in the PRISMA flow diagram (Figure 1).

**FIGURE 1:**
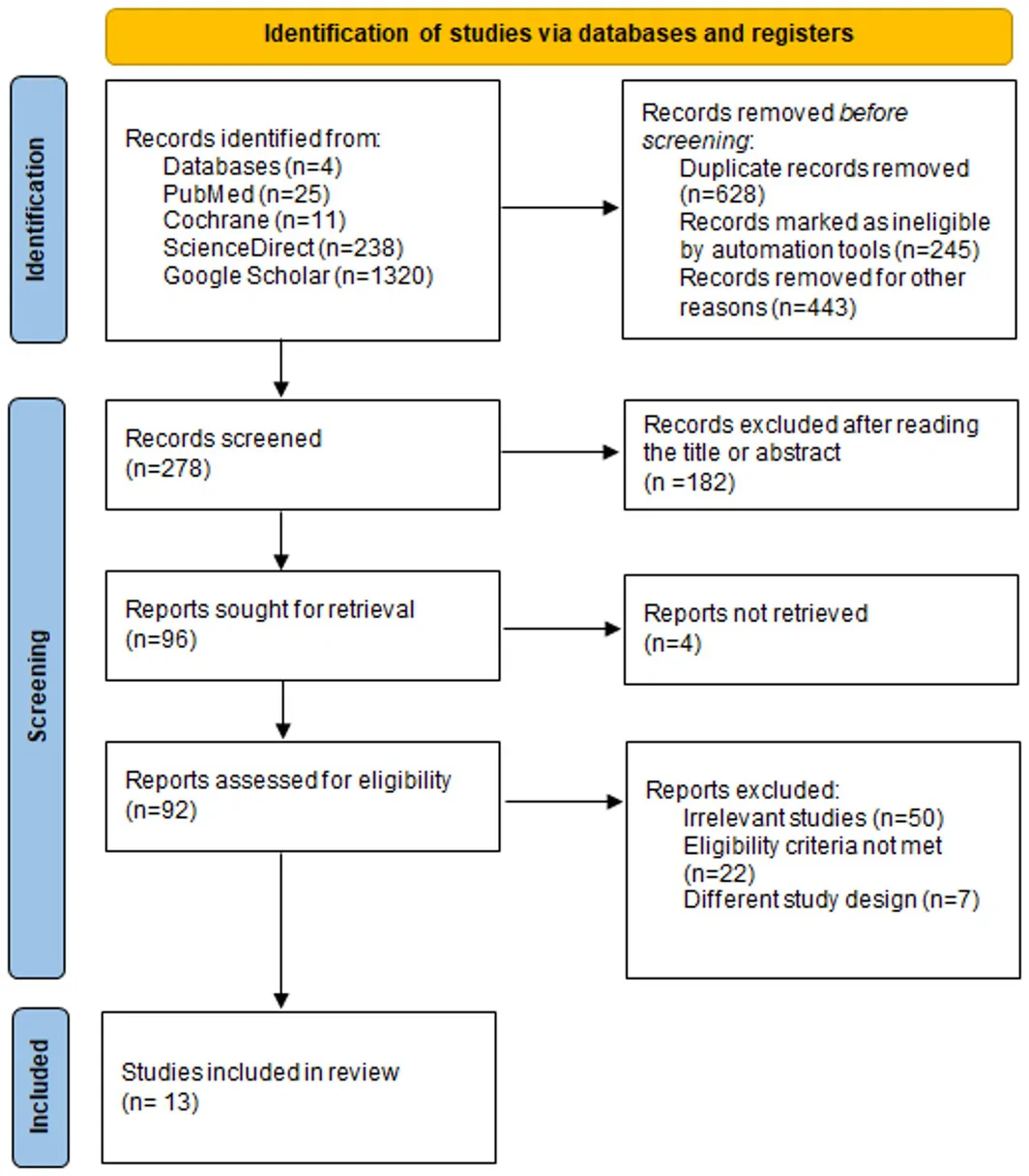
PRISMA flow diagram of the studies screened and included in the systematic review.

Among the selected studies, 12 were retrospective cohorts [4, 5, 6, 7, 8, 13, 14, 15, 16, 17, 18, 19], while 1 was an RCT [20]. A total of 2897 patients were included, comprising 954 in the RPLC group and 1943 in the MPLC group. Patients in both groups had similar ages, with a mean of 64.6 ± 10.0 years in the RPLC group and 64.9 ± 10.6 years in the MPLC group. The overall cohort included 1657 males (57.2%) and 1240 females (42.8%). The mean Body mass index (BMI) across both groups was 23.5 ± 3.3 kg/m^2^. Tumor locations varied, involving all segments of the colon and rectum. Common sites included the rectum, sigmoid colon, ascending colon, hepatic flexure, and transverse colon. Complete baseline demographic and clinical characteristics are detailed in Table S5. The reduced‐port technique was not uniform across the included studies: the RPLC arm employed either a two‐port or a three‐port configuration, frequently performed by a single surgeon assisted only by a scopist, with corresponding variation in the number and placement of ports. To clarify this variability for the reader, the number of ports used in the RPLC arm of each study is summarized in Table S5.

### 3.2 Quality Assessment of Included Studies

The quality and risk of bias of the 12 included cohort studies [4, 5, 6, 7, 8, 13, 14, 15, 16, 17, 18, 19] were assessed using the NOS, which evaluates studies across three domains: selection, comparability, and outcome. The studies demonstrated a “moderate” to “low” risk of bias, with NOS scores ranging from 6 to 9. A detailed assessment is available in Table S3. The risk of bias for the RCT was assessed using the RoB 2 tool, which identified an overall judgment of “some concerns” (Figure S1) [20].

### 3.3 Primary Outcomes

#### 3.3.1 Estimated Blood Loss

Twelve studies reported estimated blood loss in patients undergoing laparoscopic surgery with either RPLC or MPLC techniques. A statistically significant difference in estimated blood loss was observed between the two groups (MD: −13.70; 95% CI: −25.63 to −1.77; *p* = 0.02; Figure 2). Heterogeneity among studies was high (*I* ^2^ = 70%). Exclusion of both Song 2016 and Fujii 2025 [17, 20] resulted in a considerable reduction in heterogeneity (*I* ^2^ = 53%), with a more pronounced and significant effect. Funnel plot analysis was conducted to visually assess publication bias and small‐study effects. The plot appeared symmetrical and Egger's linear regression test did not reveal statistically significant funnel plot asymmetry (*p* = 0.84), indicating no significant evidence of publication bias among the included studies (Figure S2).

**FIGURE 2:**
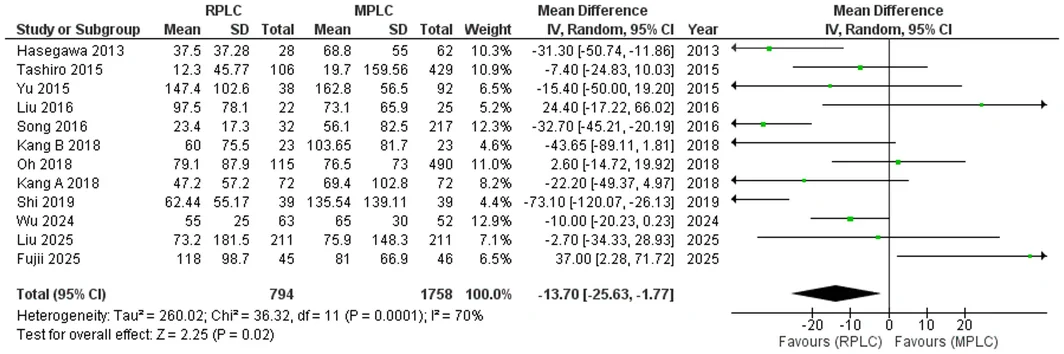
Forest plot of estimated blood loss in reduced‐port versus multi‐port laparoscopic colectomy.

#### 3.3.2 Operative Time

Twelve studies reported operative time in patients undergoing laparoscopic surgery using either RPLC or MPLC techniques. A statistically significant difference in operative time was observed between the two groups (MD: −19.98; 95% CI: −29.73 to −10.24; *p* < 0.0001; Figure 3). Heterogeneity among studies was high (*I* ^2^ = 84%). Exclusion of both Liu 2016 and Liu 2025 [8, 16] led to a considerable reduction in heterogeneity (*I* ^2^ = 58%) with a more pronounced effect size. Funnel plot analysis was conducted to visually assess publication bias and small‐study effects. The plot appeared symmetrical and Egger's linear regression test did not reveal statistically significant funnel plot asymmetry (*p* = 0.3478), indicating no significant evidence of publication bias among the included studies (Figure S3).

**FIGURE 3:**
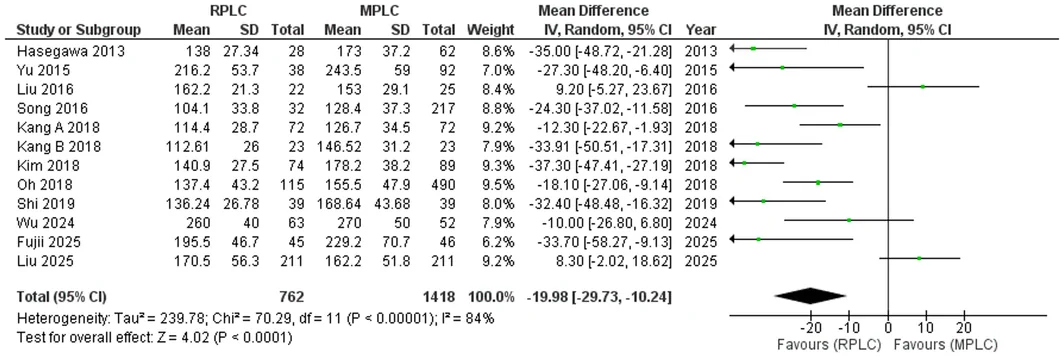
Forest plot of operative time in reduced‐port versus multi‐port laparoscopic colectomy.

#### 3.3.3 Postoperative Hospital Stay

Twelve studies reported postoperative hospital stay in patients undergoing laparoscopic surgery with either RPLC or MPLC techniques. No statistically significant difference in the time was observed between the two groups (MD: 0.65; 95% CI: −1.54 to 0.24; *p* = 0.15; Figure 4). Heterogeneity among studies was high (*I* ^2^ = 85%). After removing Hasegawa 2013 [13], heterogeneity was reduced (*I* ^2^ = 66%) and the overall result remained insignificant. Funnel plot analysis was conducted to assess the presence of publication bias for post‐operative hospital stay. Although the funnel plot showed asymmetry, Egger's linear regression test did not demonstrate statistically significant funnel plot asymmetry (*p* = 0.305) (Figure S4).

**FIGURE 4:**
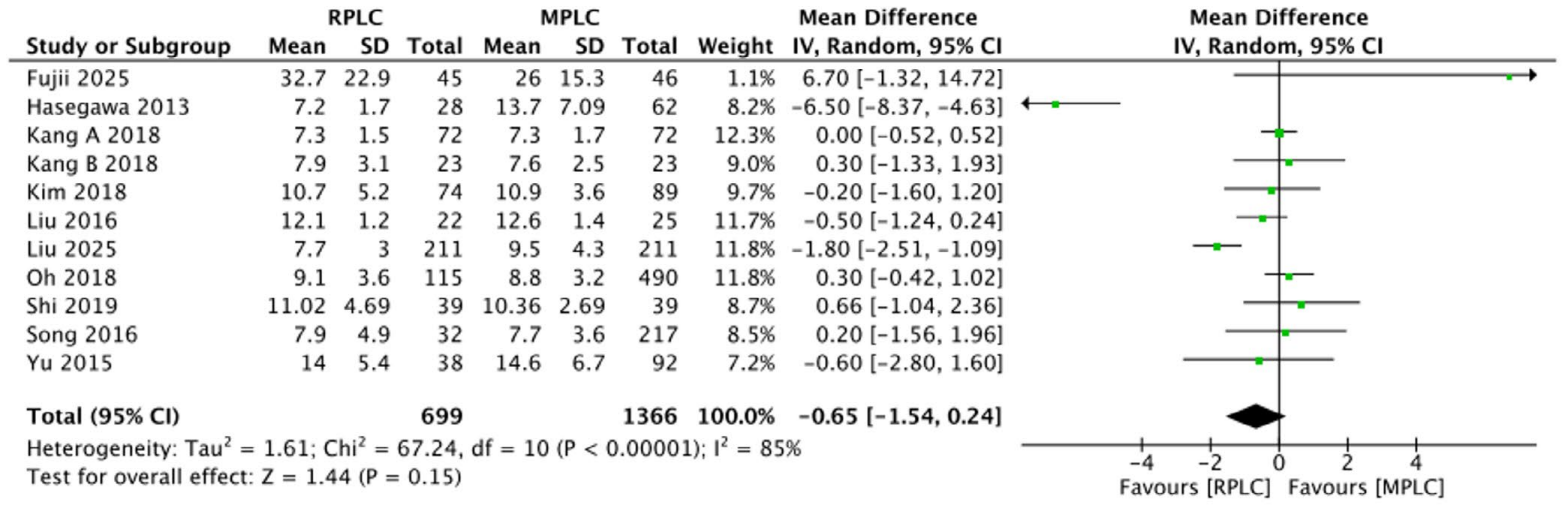
Forest plot of postoperative hospital stay in reduced‐port versus multi‐port laparoscopic colectomy.

#### 3.3.4 Harvested Lymph Nodes

Eleven studies reported the harvested lymph nodes yield in patients undergoing laparoscopic surgery with either RPLC or MPLC techniques. No statistically significant difference was observed between the two groups (MD: 0.35; 95% CI: −2.25 to 2.95; *p* = 0.79; Figure 5). Heterogeneity among studies was high (*I* ^2^ = 77%). After removing Song 2016 and Liu 2025 [8, 17], heterogeneity was reduced (*I* ^2^ = 67%), but the overall result remained insignificant. Funnel plot analysis was conducted to visually assess publication bias and small‐study effects. Although the plot showed some asymmetry, Egger's linear regression test did not reveal statistically significant funnel plot asymmetry (*p* = 0.35), indicating no significant evidence of publication bias among the included studies (Figure S5).

**FIGURE 5:**
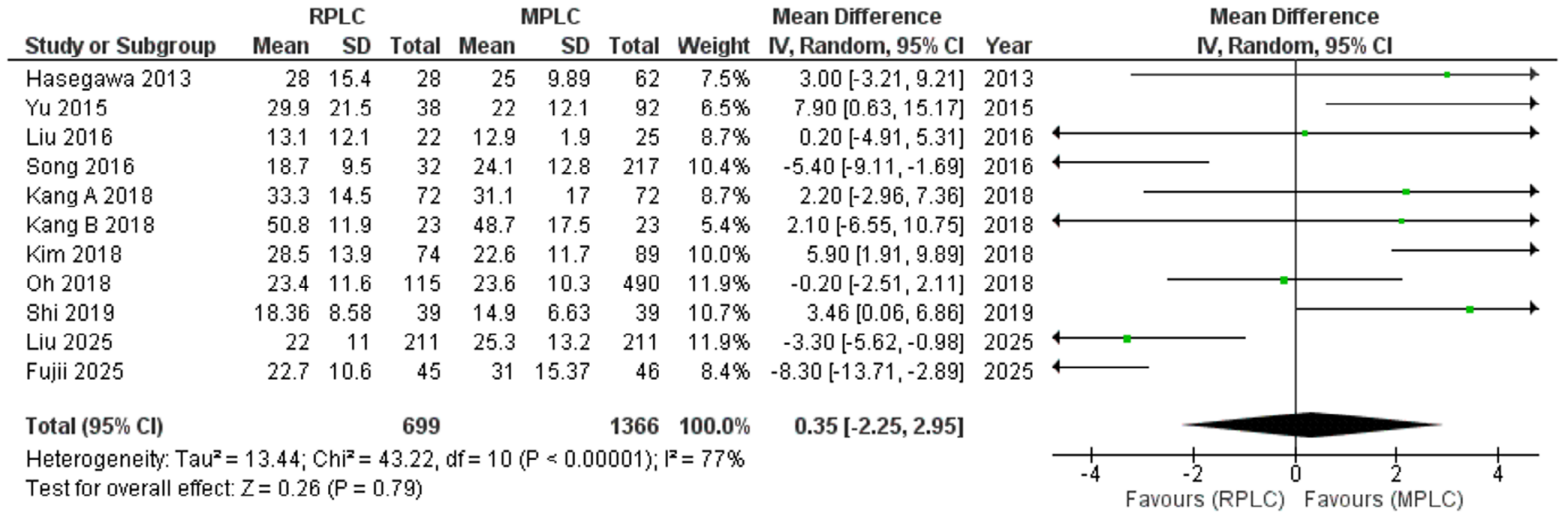
Forest plot of harvested lymph nodes yield in reduced‐port versus multi‐port laparoscopic colectomy.

#### 3.3.5 Time to First Flatus

Nine studies reported the time to first flatus passed in patients undergoing laparoscopic surgery with either RPLC or MPLC techniques. No statistically significant difference in the time to first flatus passed was observed between the two groups (MD: 0.01; 95% CI: −0.37 to −0.39; *p* = 0.96; Figure 6). Heterogeneity among studies was high (*I* ^2^ = 91%). After removing Kim 2018 [4], heterogeneity was reduced (*I* ^2^ = 84%), but the overall result remained insignificant. DOI plot showed no asymmetry (LFK index = 0.97; Figure S6), indicating no evidence of publication bias.

**FIGURE 6:**
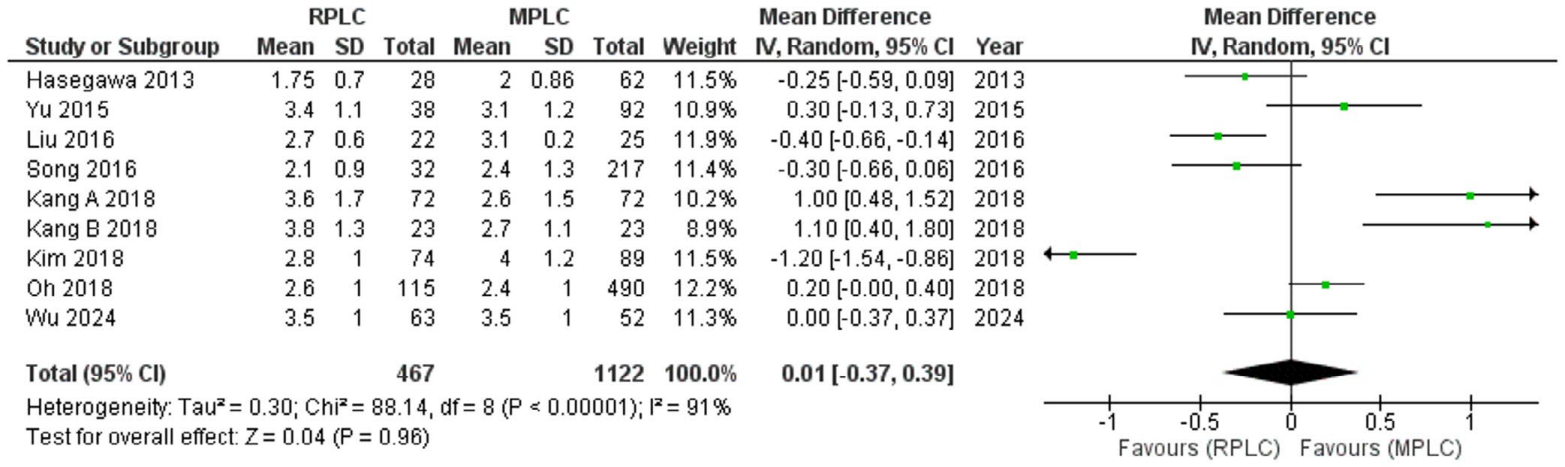
Forest plot of time to first flatus in reduced‐port versus multi‐port laparoscopic colectomy.

#### 3.3.6 Time to First Liquid Diet

Four studies reported the time to first liquid diet in patients undergoing laparoscopic surgery with either RPLC or MPLC techniques. No statistically significant difference was observed between the two groups (MD: −0.60; 95% CI: −1.55 to 0.35; *p* = 0.22; Figure S7). Heterogeneity among studies was high (*I* ^2^ = 94%). After removing Wu 2024 [5], heterogeneity was reduced (*I* ^2^ = 87), but the overall result remained insignificant. DOI plot showed minor asymmetry (LFK index = −1.16; Figure S8), suggesting the possibility of publication bias.

#### 3.3.7 Meta‐Regression

Meta‐regression revealed that a higher percentage of high‐stage tumors was significantly associated with longer operative time in the RPLC cohort (*p* = 0.0013), with each 10% increase in high‐stage tumors reducing the time‐saving benefit by approximately 6.5 min. This factor explained 52.9% of the interstudy heterogeneity (*R* ^2^ = 52.9%) (Figure S9, Table S4). However, the percentage of high‐stage tumors was not significantly associated with other outcomes, including estimated blood loss (*p* = 0.21), lymph node yield (*p* = 0.62), time to first flatus (*p* = 0.26), time to first liquid diet (*p* = 0.12), or hospital stay (*p* = 0.76) (Figures S10–S14, Table S4).

### 3.4 Secondary Outcomes

#### 3.4.1 Laparoscopic to Open Conversion

Six studies reported the rate of laparoscopic to open conversion during the surgery in patients undergoing laparoscopic surgery with either RPLC or MPLC techniques. No statistically significant difference was observed between the two groups (RR: 0.81; 95% CI: 0.42 to 1.53; *p* = 0.51; Figure S15). Heterogeneity among studies was nil (*I* ^2^ = 0%). DOI plot showed asymmetry (LFK index = −3.04; Figure S16), indicating publication bias.

#### 3.4.2 Anastomotic Bleeding Incidence

Four studies reported the incidence of anastomotic bleeding during the surgery in patients undergoing laparoscopic surgery with either RPLC or MPLC techniques. No statistically significant difference was observed between the two groups (RR: 1.04; 95% CI: 0.27 to 4.09; *p* = 0.95; Figure S17). Heterogeneity among studies was nil (*I* ^2^ = 0%). DOI plot showed minor asymmetry (LFK index = −1.21; Figure S18), suggesting the possibility of publication bias.

#### 3.4.3 Postoperative Ileus Incidence

Six studies reported the incidence of postoperative ileus in patients undergoing laparoscopic surgery using either RPLC or MPLC techniques. No statistically significant difference in the risk of postoperative ileus was observed between the two groups (RR: 0.96; 95% CI: 0.43 to 2.16; *p* = 0.92; Figure S19). Heterogeneity among studies was low (*I* ^2^ = 0%). DOI plot showed a symmetrical distribution of studies (LFK index = 0.21; Figure S20), indicating no publication bias.

#### 3.4.4 Wound Infection Incidence

Five studies reported the incidence of wound infection in patients undergoing laparoscopic surgery using either RPLC or MPLC techniques. No statistically significant difference in the risk of wound infection was observed between the two groups (RR: 0.52; 95% CI: 0.21 to 1.25; *p* = 0.14; Figure S21). Heterogeneity among studies was low (*I* ^2^ = 8%). DOI plot showed a generally asymmetrical distribution of studies (LFK index = 2.16; Figure S22), indicating significant publication bias.

#### 3.4.5 Postoperative Complication Grade 0—Clavien‐Dindo Classification

Four studies reported the postoperative complications classified as Grade 0 by the Clavien‐Dindo Classification in patients undergoing laparoscopic surgery using either RPLC or MPLC techniques. No statistically significant difference in the risk of grade 0 complications was observed between the two groups (RR: 1.01; 95% CI: 0.98–1.04; *p* = 0.63; Figure S23). Heterogeneity among studies was low (*I* ^2^ = 3%). DOI plot showed a generally symmetrical distribution of studies (LFK index = −0.57; Figure S24), indicating no publication bias.

#### 3.4.6 Postoperative Complication Grade 1—Clavien‐Dindo Classification

Five studies reported the postoperative complications classified as Grade 1 by the Clavien‐Dindo Classification in patients undergoing laparoscopic surgery using either RPLC or MPLC techniques. A statistically significant difference in the risk of grade 1 complications was observed between the two groups (RR: 1.70; 95% CI: 1.07 to 2.70; *p* = 0.02; Figure S25). Heterogeneity among studies was nil (*I* ^2^ = 0%). DOI plot showed a generally symmetrical distribution of studies (LFK index = 0.68; Figure S26), indicating no publication bias.

#### 3.4.7 Postoperative Complication Grade 2—Clavien‐Dindo Classification

Seven studies reported the postoperative complications classified as Grade 2 by the Clavien‐Dindo Classification in patients undergoing laparoscopic surgery using either RPLC or MPLC techniques. No statistically significant difference in the risk of grade 2 complications was observed between the two groups (RR: 0.81; 95% CI: 0.40 to 1.64; *p* = 0.55; Figure S27). Heterogeneity among studies was low (*I* ^2^ = 27%). DOI plot showed asymmetrical distribution of studies (LFK index = −2.01; Figure S28), indicating significant publication bias.

#### 3.4.8 Postoperative Complication Grade 3—Clavien‐Dindo Classification

Six studies reported the postoperative complications classified as Grade 3 by the Clavien‐Dindo Classification in patients undergoing laparoscopic surgery using either RPLC or MPLC techniques. No statistically significant difference in the risk of grade 3 complications was observed between the two groups (RR: 0.87; 95% CI: 0.36 to 2.06; *p* = 0.75; Figure S29). Heterogeneity among studies was nil (*I* ^2^ = 0%). DOI plot showed a generally symmetrical distribution of studies (LFK index = 0.83; Figure S30), indicating no publication bias.

#### 3.4.9 Postoperative Complication Grade 4—Clavien‐Dindo Classification

Two studies reported the postoperative complications classified as Grade 4 by the Clavien‐Dindo Classification in patients undergoing laparoscopic surgery using either RPLC or MPLC techniques. No statistically significant difference in the risk of grade 4 complications was observed between the two groups (RR: 4.15; 95% CI: 0.47 to 37.0; *p* = 0.20; Figure S31). Heterogeneity among studies was nil (*I* ^2^ = 0%). DOI plot showed minor asymmetry (LFK index = −1.8; Figure S32), suggesting the possibility of publication bias.

## 4 Discussion

This review demonstrates that RPLC has a comparable safety profile to multi‐port techniques, with modest intraoperative benefits. While SAGES guidelines support minimally invasive surgery for resectable CRC, they do not specify a preferred port configuration [21]. Our findings support RPLC as a viable alternative, offering similar perioperative and short‐term oncologic surrogate outcomes (notably lymph node yield, in the absence of recurrence and survival data).

The observed benefits of RPLC, particularly in terms of lower estimated blood loss and shorter operative time, may be explained by several technical and procedural factors. RPLC typically utilizes fewer port sites with optimized placement, which may reduce tissue trauma and vascular injury during entry and dissection. A study conducted by Song et al. reported that blood loss was significantly lower in the RPLC group (24 mL) compared to MPLC (56 mL), highlighting the role of streamlined port placement and potentially less manipulation in reducing intraoperative bleeding [17].

Similarly, shorter operative times may result from more efficient instrument handling and reduced time spent on port insertion and closure. Although a reduced number of incisions might be expected to lower the rate of minor wound‐related events, our pooled analysis did not support this assumption. On the contrary, the risk of Clavien–Dindo grade 1 complications was significantly higher in the RPLC group (RR: 1.70; 95% CI: 1.07–2.70; *p* = 0.02), whereas wound infection, anastomotic bleeding, and grade 2–4 complications did not differ significantly between groups. This apparent paradox should be interpreted with caution: it may reflect the greater technical demand of operating through fewer ports, more attentive ascertainment and reporting of minor events in the reduced‐port cohort, or residual confounding inherent to the predominantly retrospective source studies, rather than a true protective effect of either approach. However, our meta‐regression analysis showed that this efficiency advantage diminishes with increasing tumor complexity. In high‐stage cases, the technical difficulty and need for more careful dissection likely outweigh the procedural simplicity of reduced‐port access. This suggests that tumor staging plays a key role in influencing operative performance across both surgical approaches.

The substantial statistical heterogeneity observed for operative time (*I* ^2^ = 84%) and estimated blood loss (*I* ^2^ = 70%) warrants careful clinical interpretation. Beyond the high‐stage tumor proportion identified in meta‐regression, this heterogeneity most likely reflects clinical and methodological diversity among the included studies. The studies spanned more than a decade (2013–2025), multiple countries, and heterogeneous institutional practices, and differed in case mix (proportion of rectal versus colonic resections and of right‐ versus left‐sided procedures), in perioperative and enhanced‐recovery management protocols, and in the specific reduced‐port techniques employed. Differences in surgeon experience and in the learning curve associated with reduced‐port access are an additional plausible source of variability, particularly for operative time. These factors, rather than the surgical approach alone, may substantially influence operative complexity and perioperative outcomes, and they should be borne in mind when interpreting the pooled estimates.

Direct comparative data on RPLC remain limited. In the absence of prior meta‐analyses specific to this technique, we cautiously compared our findings to meta‐analyses on single‐incision laparoscopic colectomy (SILC). Brockhaus et al. reported similar oncologic outcomes for SILC versus conventional laparoscopy, although SILC had longer operative durations [22]. Unlike SILC, RPLC allows better triangulation and instrument maneuverability, which may explain the shorter operative times observed in our analysis. Furthermore, our results align with meta‐analyses on reduced‐port techniques in gastric surgery, reinforcing the broader applicability of reduced‐port strategies in gastrointestinal surgery [23].

This meta‐analysis has several strengths. It focused exclusively on comparing RPLC and MPLC for CRC, ensuring clinical relevance while excluding unrelated techniques, such as SIL. The large pooled sample of 2897 patients from 13 studies provides strong statistical power, and the inclusion of studies up to June 2025 reflects current surgical practices. Study quality was rigorously assessed using the Newcastle‐Ottawa Scale and Cochrane RoB 2.0. Advanced statistical methods, including random‐effects modeling, leave‐one‐out sensitivity analysis, and meta‐regression, were used to reduce heterogeneity and explore effect modifiers. Publication bias was evaluated using Egger's test and DOI plots, which enhance the study's transparency and credibility. Notably, meta‐regression identified tumor stage as a significant modifier of operative time, with higher‐stage tumors diminishing the efficiency benefit typically associated with reduced‐port surgery. This single factor accounted for over 50% of the between‐study heterogeneity, emphasizing its clinical importance in shaping surgical outcomes.

Despite these strengths, several significant limitations must be acknowledged. The majority of included studies were retrospective cohort designs, which inherently risk selection bias and confounding. In particular, reduced‐port procedures may have been preferentially offered by more experienced surgeons to carefully selected patients with lower‐risk disease, introducing a substantial risk of selection bias that could favor RPLC. Although meta‐regression was used to explore the influence of high‐stage tumors, several important determinants of operative difficulty—including body mass index and obesity, T4 disease, prior abdominal surgery, and other markers of surgical complexity—were inconsistently reported in the source studies and could not be adjusted for. The apparent intraoperative advantages of RPLC should therefore be interpreted with corresponding caution. Substantial heterogeneity was observed across outcomes, likely due to differences in surgical expertise, perioperative protocols, and port configurations. A further methodological limitation concerns the heterogeneous definition of RPLC itself: two‐port, three‐port, and solo‐surgeon techniques, together with variation in scopist involvement and in the number and placement of ports, were pooled under a single category. Because these technical differences may directly affect operative time and estimated blood loss, the corresponding pooled estimates should be regarded as approximations across a spectrum of reduced‐port techniques rather than as a single, uniformly defined intervention. Injury and complication definitions varied, as did the quality of reporting. Most studies lacked long‐term oncologic follow‐up, which limited conclusions regarding recurrence and survival. The included studies reported predominantly short‐term perioperative endpoints, and few provided recurrence, disease‐free, or overall survival data; the present analysis therefore cannot establish the long‐term oncologic safety of RPLC. For the same reason, the comparable lymph node yields observed between groups should not be equated with equivalent oncologic adequacy, as nodal harvest is only a surrogate marker and does not, by itself, reflect recurrence or survival. In addition, conversion from RPLC to multi‐port surgery (port addition) and its underlying reasons were not consistently reported across the included studies, precluding a reliable pooled estimate of this clinically relevant event; likewise, tumor size could not be evaluated as a moderator of operative time alongside tumor stage, owing to inconsistent reporting of size as a continuous variable. The certainty of evidence was rated as very low due to these limitations, and the absence of standardized training for RPLC means that results may not be generalizable to all surgical settings. Furthermore, the prevalence of high‐stage tumors influenced operative time outcomes, complicating interpretation without patient‐level data. Moreover, the certainty of evidence, as assessed via the GRADE framework, was generally “very low” to “low” across key outcomes. This was primarily due to the non‐randomized study designs, serious heterogeneity among studies, and indications of publication bias. These factors limit confidence in our effect estimates and underscore the need for high‐quality RCTs in this area.

This study supports the growing viability of RPLC as a safe, minimally invasive option for CRC surgery. Importantly, it may offer specific benefits in reducing operative time and blood loss without compromising core outcomes. Given that RPLC uses standard laparoscopic instruments with minor modifications, it is adaptable to low‐ and middle‐income countries (LMICs), where cost and infrastructure are limiting factors. This potential, however, should not be overstated. RPLC is technically demanding, and because it requires advanced laparoscopic skill its outcomes may not be reproducible across all settings. Successful implementation depends less on the avoidance of dedicated robotic equipment than on the availability of appropriate surgical expertise, a defined learning curve, and structured training and institutional support; adoption in resource‐limited settings should therefore proceed alongside such training and proctoring rather than be assumed to follow from the lower equipment requirement alone. Future research should focus on high‐quality RCTs with standardized outcome reporting and adequate long‐term follow‐up, including oncologic endpoints such as recurrence, disease‐free survival, and overall survival, which were largely unavailable in the current evidence base. Additionally, patient‐centered outcomes, such as postoperative pain, recovery time, and quality of life, should be systematically evaluated. Regional surgical societies might consider developing shared training modules and registries to enhance technique standardization and data collection. With growing climate‐related strain on healthcare systems and surgical resources, adopting efficient, less resource‐intensive techniques like RPLC could improve resilience in surgical care delivery.

## 5 Conclusion

This systematic review and meta‐analysis of 13 studies compared RPLC and MPLC in the context of CRC surgery. RPLC significantly reduced operative time and estimated blood loss without compromising postoperative outcomes, including hospital stay, lymph node yield, and complication rates. However, because most of the included studies were retrospective and substantial heterogeneity was present for several key outcomes, these findings should be interpreted as supporting the feasibility and short‐term safety of RPLC as an alternative to MPLC, rather than as establishing its superiority. Future high‐quality RCTs with standardized techniques and longer follow‐up are required to confirm these results, to establish the long‐term oncologic safety of RPLC (including recurrence and survival) and guide clinical practice effectively. The choice of laparoscopic approach should consider the surgeon's expertise, the patient's characteristics, and the availability of resources.

## Author Contributions

**Saad Ashraf:** data curation, project administration, formal analysis. **Hamza Irfan:** formal analysis, software. **Um E. Abiha Batool:** data curation, writing – review and editing. **Muhammad Shahmeer Ullah Shah:** data curation, writing – original draft. **Ayesha Shaukat:** conceptualization, data curation, writing – original draft. **Santosh Sah:** visualization, writing – review and editing. **Omer Bin Khalid Jamil:** writing – review and editing. **Muhammad Zain Ul Haq:** writing – review and editing. **Aymar Akilimali:** writing – review and editing. **Yumna Khabir:** methodology, writing – original draft.

## Funding

The authors have nothing to report.

## Ethics Statement

This article is based on previously conducted studies and does not contain any new studies with human participants or animals performed by any of the authors.

## Consent

The authors have nothing to report.

## Conflicts of Interest

The authors declare no conflicts of interest.

## Supporting information


**Figure S1:** Risk of bias summary plot (A) and traffic light plot (B) for the included trial.
**Figure S2:** Funnel plot analysis of estimated blood loss in reduced‐port versus multi‐port laparoscopic colectomy.
**Figure S3:** Funnel plot analysis of operative time in reduced‐port versus multi‐port laparoscopic colectomy.
**Figure S4:** Funnel plot analysis of postoperative hospital stay in reduced‐port versus multi‐port laparoscopic colectomy.
**Figure S5:** Funnel plot analysis of harvested lymph nodes yield in reduced‐port versus multi‐port laparoscopic colectomy.
**Figure S6:** DOI plot of time to first flatus in reduced‐port versus multi‐port laparoscopic colectomy.
**Figure S7:** Forest plot of time to first liquid diet in reduced‐port versus multi‐port laparoscopic colectomy.
**Figure S8:** DOI plot of time to first liquid diet in reduced‐port versus multi‐port laparoscopic colectomy.
**Figure S9:** Meta‐regression plot showing the association between high‐stage tumor (%) and operative time.
**Figure S10:** Meta‐regression plot showing the association between high‐stage tumor (%) and estimated blood loss.
**Figure S11:** Meta‐regression plot showing the association between high‐stage tumor (%) and harvested lymph nodes.
**Figure S12:** Meta‐regression plot showing the association between high‐stage tumor (%) and time to first flatus.
**Figure S13:** Meta‐regression plot showing the association between high‐stage tumor (%) and time to first liquid diet.
**Figure S14:** Meta‐regression plot showing the association between high‐stage tumor (%) and postoperative hospital stay.
**Figure S15:** Forest plot of laparoscopic to open conversion rate in reduced‐port versus multi‐port laparoscopic colectomy.
**Figure S16:** DOI plot of laparoscopic to open conversion rate in reduced‐port versus multi‐port laparoscopic colectomy.
**Figure S17:** Forest plot of anastomotic bleeding incidence in reduced‐port versus multi‐port laparoscopic colectomy.
**Figure S18:** DOI plot of anastomotic bleeding incidence in reduced‐port versus multi‐port laparoscopic colectomy.
**Figure S19:** Forest plot of postoperative ileus incidence in reduced‐port versus multi‐port laparoscopic colectomy.
**Figure S20:** DOI plot of postoperative ileus incidence in reduced‐port versus multi‐port laparoscopic colectomy.
**Figure S21:** Forest plot of wound infection incidence in reduced‐port versus multi‐port laparoscopic colectomy.
**Figure S22:** DOI plot of wound infection incidence in reduced‐port versus multi‐port laparoscopic colectomy.
**Figure S23:** Forest plot of postoperative complications classified as Grade 0 Clavien‐dindo Classification in reduced‐port versus multi‐port laparoscopic colectomy.
**Figure S24:** DOI plot of postoperative complications classified as Grade 0 Clavien‐dindo Classification in reduced‐port versus multi‐port laparoscopic colectomy.
**Figure S25:** Forest plot of postoperative complications classified as Grade 1 Clavien‐dindo Classification in reduced‐port versus multi‐port laparoscopic colectomy.
**Figure S26:** DOI plot of postoperative complications classified as Grade 1 Clavien‐dindo Classification in reduced‐port versus multi‐port laparoscopic colectomy.
**Figure S27:** Forest plot of postoperative complications classified as Grade 2 Clavien‐dindo Classification in reduced‐port versus multi‐port laparoscopic colectomy.
**Figure S28:** DOI plot of postoperative complications classified as Grade 2 Clavien‐dindo Classification in reduced‐port versus multi‐port laparoscopic colectomy.
**Figure S29:** Forest plot of postoperative complications classified as Grade 3 Clavien‐dindo Classification in reduced‐port versus multi‐port laparoscopic colectomy.
**Figure S30:** DOI plot of postoperative complications classified as Grade 3 Clavien‐dindo Classification in reduced‐port versus multi‐port laparoscopic colectomy.
**Figure S31:** Forest plot of postoperative complications classified as Grade 4 Clavien‐dindo Classification in reduced‐port versus multi‐port laparoscopic colectomy.
**Figure S32:** DOI plot of postoperative complications classified as Grade 4 Clavien‐dindo Classification in reduced‐port versus multi‐port laparoscopic colectomy.
**Table S1:** Detailed search strategy used for each database (PubMed, Google Scholar, ScienceDirect, Cochrane) in the systematic review.
**Table S2:** Quality of evidence for outcomes using the GRADE (Grading of Recommendations Assessment, Development, and Evaluation) framework.
**Table S3:** Newcastle‐Ottawa scale ratings for included studies.
**Table S4:** Meta‐regression evaluating the impact of high‐stage tumor (%) on surgical outcomes.

**Table S5:** Baseline characteristics of the included studies.

## Data Availability

The authors have nothing to report.
