# Supplementary material for: Reduced‐Port Versus Multi‐Port Laparoscopic Colectomy for Colorectal Cancer: A Systematic Review, Meta‐Analysis, and Meta‐Regression
Source: Ann Gastroenterol Surg. 2026 Aug 18;10(6):1712–21. doi: 10.1002/ags3.70264 (PMC13731019; doi:10.1002/ags3.70264)
Supplement: Supplementary file 1 [file AGS3-10-1712-s001.docx]

**SUPPLEMENTARY FILE**

**Title: Reduced-Port vs. Multi-Port Laparoscopic Colectomy for Colorectal Cancer: A Systematic Review, Meta-Analysis, and Meta-Regression**

**Authors:**

Ayesha Shaukat, MBBS^1^; Saad Ashraf, MBBS^1^; Hamza Irfan, MBBS^2^; Muhammad Shahmeer Ullah Shah, MBBS^1^; Yumna Khabir, MBBS^1^; Santosh Sah, MBBS^1^; Um E Abiha Batool, MBBS^1^; Muhammad Zain Ul Haq, MBBS^1^; Omer Bin Khalid Jamil, MBBS, FCPS (Surgery), FACS^3^; Aymar Akilimali, MD^4^

**Affiliations:**

^1^ Department of Medicine, Dow University of Health Sciences, Karachi, Pakistan

^2^ Department of Medicine, Shaikh Khalifa Bin Zayed Al Nahyan Medical and Dental College, Lahore, Pakistan

^3^ Assistant Professor Surgery, Department of Surgery, Dow University of Health Sciences, Karachi, Pakistan

^4^ Medical Research Circle, Goma, Democratic Republic of the Congo

**Corresponding Author: Aymar Akilimali, MD**

**Affiliation:** Department of research, Medical Research Circle (MedReC), Goma, Democratic Republic of the Congo

**Email address:** [aymarakilimali@gmail.com](mailto:aymarakilimali@gmail.com); **ORCID:** 0000-0001-9393-1215

| **Databases** | **Search Strategy** |
| --- | --- |
| **PubMed** | ("Colectomy"[MeSH Terms] OR colectomy[tiab] OR hemicolectomy[tiab] OR "right colectomy"[tiab] OR "right hemicolectomy"[tiab] OR "colon resection"[tiab]) AND ("Laparoscopy"[MeSH Terms] OR laparoscop*[tiab] OR "minimally invasive surgery"[tiab] OR "laparoscopic surgery"[tiab]) AND ("two port"[tiab] OR "2 port"[tiab] OR "2-port"[tiab] OR "three port"[tiab] OR "three-port"[tiab] OR "3 port"[tiab] OR "3-port"[tiab] OR "reduced port"[tiab] OR "triad port"[tiab] OR RPLC[tiab]) AND ("four port"[tiab] OR "4 port"[tiab] OR "4-port"[tiab] OR "five port"[tiab] OR "5-port"[tiab] OR "multi-port"[tiab] OR "conventional laparoscopic"[tiab] OR MPLC[tiab]) AND ("Colonic Neoplasms"[MeSH Terms] OR "colon cancer"[tiab] OR "colorectal cancer"[tiab] OR "right-sided colon cancer"[tiab] OR "colon adenocarcinoma"[tiab] OR "colonic neoplasm*"[tiab]) |
| **Google Scholar** | "right hemicolectomy" OR "right colectomy" OR "colon resection" AND ("laparoscopic" OR "minimally invasive surgery") AND ("2 port" OR "2-port" OR "two port" OR "3 port" OR "3-port" OR "three port" OR "reduced port" OR RPLC) AND ("4 port" OR "4-port" OR "five port" OR "5-port" OR "multi-port" OR MPLC OR "conventional laparoscopic") AND ("colon cancer" OR "colorectal cancer" OR "colonic neoplasms") |
| **ScienceDirect** | TITLE-ABSTR-KEY("right colectomy" OR "right hemicolectomy" OR "colon resection") AND TITLE-ABSTR-KEY("laparoscopic" OR "minimally invasive surgery") AND TITLE-ABSTR-KEY("2 port" OR "2-port" OR "three port" OR "3 port" OR "reduced port" OR RPLC) AND TITLE-ABSTR-KEY("4 port" OR "4-port" OR "five port" OR "5 port" OR "multi-port" OR MPLC OR "conventional laparoscopic") AND TITLE-ABSTR-KEY("colon cancer" OR "colorectal cancer" OR "colonic neoplasm") |
| **Cochrane Library** | (colectomy OR "colon resection" OR "right colectomy" OR "right hemicolectomy") AND (laparoscopic OR "minimally invasive") AND ("2 port" OR "2-port" OR "three port" OR "3-port" OR "reduced port" OR RPLC) AND ("4 port" OR "4-port" OR "five port" OR "5-port" OR "multi-port" OR MPLC OR "conventional laparoscopic") AND ("colon cancer" OR "colorectal cancer" OR "colonic neoplasm") |

**Supplementary Table 1:** Detailed search strategy used for each database (PubMed, Google Scholar, ScienceDirect, Cochrane) in the systematic review.

| **Certainty assessment** | | | | | | | **№ of patients** | | **Effect** | | **Certainty** | **Importance** |
| --- | --- | --- | --- | --- | --- | --- | --- | --- | --- | --- | --- | --- |
| **№ of studies** | **Study design** | **Risk of bias** | **Inconsistency** | **Indirectness** | **Imprecision** | **Other considerations** | **Reduced-port laparoscopic colectomy (RPLC)** | **Multi-port laparoscopic colectomy (MPLC)** | **Relative (95% CI)** | **Absolute (95% CI)** |  |  |
| **Operative time (assessed with: Minutes)** | | | | | | | | | | | | |
| 12 | non-randomised studies | not serious | serious^a^ | not serious | not serious | none | 762 | 1418 | - | MD **19.98 minutes fewer** (29.73 fewer to 10.24 fewer) | ⨁◯◯◯ Very low^a^ | CRITICAL |
| **Estimated blood loss (assessed with: Milliliters (mL))** | | | | | | | | | | | | |
| 12 | non-randomised studies | not serious | serious^b^ | not serious | not serious | none | 794 | 1758 | - | MD **13.7 mL fewer** (25.63 fewer to 1.77 fewer) | ⨁◯◯◯ Very low^b^ | CRITICAL |
| **Postoperative Hospital Stay (assessed with: Days)** | | | | | | | | | | | | |
| 12 | non-randomised studies | not serious | very serious^c^ | not serious | serious^d^ | none | 805 | 1795 | - | MD **0.84 days more** (0.63 fewer to 2.31 more) | ⨁◯◯◯ Very low^c,d^ | CRITICAL |
| **Harvested Lymph Nodes (assessed with: Number of lymph nodes)** | | | | | | | | | | | | |
| 11 | non-randomised studies | not serious | serious^e^ | not serious | serious^f^ | none | 699 | 1366 | - | MD **0.35 nodes higher** (2.25 lower to 2.95 higher) | ⨁◯◯◯ Very low^e,f^ | CRITICAL |
| **Time to First Flatus (assessed with: Days)** | | | | | | | | | | | | |
| 9 | non-randomised studies | not serious | serious^g^ | not serious | serious^h^ | none | 467 | 1122 | - | MD **0.01 days higher** (0.37 lower to 0.39 higher) | ⨁◯◯◯ Very low^g,h^ | IMPORTANT |
| **Laparoscopic to Open Conversion (assessed with: Events/Total)** | | | | | | | | | | | | |
| 6 | non-randomised studies | not serious | not serious | not serious | not serious | publication bias strongly suspected^i^ | 12/336 (3.6%) | 35/892 (3.9%) | **RR 0.81** (0.42 to 1.53) | **7 fewer per 1,000** (from 23 fewer to 21 more) | ⨁◯◯◯ Very low^i^ | CRITICAL |
| **Postoperative Complication Grade 1– Clavien-Dindo Classification (assessed with: Events/Total)** | | | | | | | | | | | | |
| 5 | non-randomised studies | not serious | not serious | not serious | not serious | none | 32/287 (11.1%) | 41/716 (5.7%) | **RR 1.70** (1.07 to 2.70) | **40 more per 1,000** (from 4 more to 97 more) | ⨁⨁◯◯ Low | CRITICAL |

**CI:** confidence interval; **MD:** mean difference; **RR:** risk ratio

a. Heterogeneity was high (I² = 84%), likely due to variation in operative protocols and surgeon experience. Direction of effect consistent across studies. Sensitivity analysis reduced I² to 58%.

b. Heterogeneity was high (I² = 70%), likely due to variation in operative protocols and surgeon experience. Direction of effect consistent across studies. Sensitivity analysis reduced I² to 53%.

c. Heterogeneity was very high (I² = 95%) and direction of effect was inconsistent across studies. Some favored RPLC, others MPLC, and some showed no difference. Sensitivity analysis reduced I² to 66%.

d. The 95% confidence interval (-0.63 to 2.31 days) includes both a potential benefit and harm, and the result is not statistically significant (p = 0.26). This level of uncertainty in direction and clinical impact warrants downgrading by 1 level.

e. Substantial heterogeneity across studies (I² = 77%), possibly due to differences in surgical technique and pathology reporting. Sensitivity analysis reduced I² to 67%,

f. Effect size small and CI (-2.25 to 2.95) crosses the null with no statistical significance (p = 0.79).

g. Heterogeneity was very high (I² = 91%), likely due to differences in definitions of flatus timing, perioperative management, and surgical protocols across studies.

h. The 95% CI for the pooled MD (0.01 [-0.37, 0.39]) includes both clinically meaningful benefit and harm; null effect cannot be excluded.

i. Doi plot showed major asymmetry (LFK index = -3.04), indicating likely publication bias.

**Supplementary Table 2:** Quality of evidence for outcomes using the GRADE (Grading of Recommendations Assessment, Development, and Evaluation) framework.

| **Study** | **Representativeness of the exposed cohort (1)** | **Selection of the non-exposed cohort (1)** | **Ascertainment of exposure (1)** | **Outcome not present at start (1)** | **Comparability of cohorts (2)** | **Assessment of outcome (1)** | **Follow-up long enough (1)** | **Adequacy of follow-up (1)** | **Total score (9)** |
| --- | --- | --- | --- | --- | --- | --- | --- | --- | --- |
| Kim et al 2018 | - | ☆ | ☆ | ☆ | ☆ | ☆ | ☆ | ☆ | **7** |
| Shi et al 2019 | - | ☆ | ☆ | ☆ | ☆☆ | ☆ | ☆ | - | **7** |
| Zhang et al 2021 | - | ☆ | ☆ | ☆ | - | ☆ | ☆ | ☆ | **6** |
| Tashiro et al 2015 | ☆ | ☆ | ☆ | ☆ | - | ☆ | ☆ | - | **6** |
| Hasegawa et al 2013 | ☆ | ☆ | ☆ | ☆ | ☆ | ☆ | ☆ | - | **7** |
| Liu et al 2025 | ☆ | ☆ | ☆ | ☆ | ☆☆ | ☆ | ☆ | ☆ | **9** |
| Liu et al 2016 | - | ☆ | ☆ | ☆ | - | ☆ | ☆ | ☆ | **6** |
| Song et al 2016 | ☆ | ☆ | ☆ | ☆ | - | ☆ | ☆ | ☆ | **7** |
| Oh et al 2018 | ☆ | ☆ | ☆ | ☆ | - | ☆ | ☆ | ☆ | **7** |
| Yu et al 2015 | ☆ | ☆ | ☆ | ☆ | ☆☆ | ☆ | ☆ | ☆ | **9** |
| Kang et al 2018 | ☆ | ☆ | ☆ | ☆ | ☆☆ | ☆ | ☆ | ☆ | **9** |
| Wu et al 2024 | - | ☆ | ☆ | ☆ | ☆ | ☆ | ☆ | ☆ | **7** |

**Supplementary Table 3:** Newcastle-Ottawa scale ratings for included studies.

| **Outcome** | **Slope (β₁)** | **95% CI** | **p-value** | **R² (%)** | **QM** |
| --- | --- | --- | --- | --- | --- |
| Operative Time (min) | +0.648 | [0.252, 1.044] | 0.0013 | 52.9 | 10.27 |
| Estimated Blood Loss (mL) | +0.469 | [–0.268, 1.207] | 0.2121 | 2.0 | 1.56 |
| Time to Liquid Diet (days) | –0.059 | [–0.134, 0.015] | 0.1178 | 36.3 | 2.45 |
| Time to First Flatus (days) | –0.013 | [–0.035, 0.009] | 0.2582 | 2.1 | 1.28 |
| Hospital Stay (days) | +0.047 | [–0.256, 0.350] | 0.7604 | 0.0 | 0.09 |
| Lymph Nodes Harvested (n) | –0.042 | [–0.211, 0.126] | 0.6227 | 0.0 | 0.24 |

**Supplementary Table 4:** Meta-Regression evaluating the impact of high-stage tumor (%) on surgical outcomes.

(**β₁**: Slope Coefficient; **CI**: Confidence interval; **p-value**: Probability value; **R² (%)**: Proportion of between-study variance explained; **QM**: Test statistic for the moderator; **min**: Minutes; **mL**: Milliliter; **n**: Number)

**B**

**A**

**Supplementary Figure 1:** Risk of bias summary plot (A) and traffic light plot (B) for the included trial.

**Supplementary Figure 2:** Funnel plot analysis of estimated blood loss in reduced-port versus multi-port laparoscopic colectomy.

**Supplementary Figure 3:** Funnel plot analysis of operative time in reduced-port versus multi-port laparoscopic colectom y.

**Supplementary Figure 4:** Funnel plot analysis of postoperative hospital stay in reduced-port versus multi-port laparoscopic colectomy.

**Supplementary Figure 5:** Funnel plot analysis of harvested lymph nodes yield in reduced-port versus multi-port laparoscopic colectomy.

**Supplementary Figure 6:** DOI plot of time to first flatus in reduced-port versus multi-port laparoscopic colectomy.

**Supplementary Figure 7:** Forest plot of time to first liquid diet in reduced-port versus multi-port laparoscopic colectomy.

**Supplementary Figure 8:** DOI plot of time to first liquid diet in reduced-port versus multi-port laparoscopic colectomy.

**Supplementary Figure 9:** Meta-regression plot showing the association between high-stage tumor (%) and operative time.

**Supplementary Figure 10:** Meta-regression plot showing the association between high-stage tumor (%) and estimated blood loss.

**Supplementary Figure 11:** Meta-regression plot showing the association between high-stage tumor (%) and harvested lymph nodes.

**Supplementary Figure 12:** Meta-regression plot showing the association between high-stage tumor (%) and time to first flatus.

**Supplementary Figure 13:** Meta-regression plot showing the association between high-stage tumor (%) and time to first liquid diet.

**Supplementary Figure 14:** Meta-regression plot showing the association between high-stage tumor (%) and postoperative hospital stay.

**Supplementary Figure 15:** Forest plot of laparoscopic to open conversion rate in reduced-port versus multi-port laparoscopic colectomy.

**Supplementary Figure 16:** DOI plot of laparoscopic to open conversion rate in reduced-port versus multi-port laparoscopic colectomy.

**Supplementary Figure 17:** Forest plot of anastomotic bleeding incidence in reduced-port versus multi-port laparoscopic colectomy.

**Supplementary Figure 18:** DOI plot of anastomotic bleeding incidence in reduced-port versus multi-port laparoscopic colectomy.

**Supplementary Figure 19:** Forest plot of postoperative ileus incidence in reduced-port versus multi-port laparoscopic colectomy.

**Supplementary Figure 20:** DOI plot of postoperative ileus incidence in reduced-port versus multi-port laparoscopic colectomy.

**Supplementary Figure 21:** Forest plot of wound infection incidence in reduced-port versus multi-port laparoscopic colectomy.

**Supplementary Figure 22:** DOI plot of wound infection incidence in reduced-port versus multi-port laparoscopic colectomy.

**Supplementary Figure 23:** Forest plot of postoperative complications classified as Grade 0 Clavien-dindo Classification in reduced-port versus multi-port laparoscopic colectomy.

**Supplementary Figure 24:** DOI plot of postoperative complications classified as Grade 0 Clavien-dindo Classification in reduced-port versus multi-port laparoscopic colectomy.

**Supplementary Figure 25:** Forest plot of postoperative complications classified as Grade 1 Clavien-dindo Classification in reduced-port versus multi-port laparoscopic colectomy.

**Supplementary Figure 26:** DOI plot of postoperative complications classified as Grade 1 Clavien-dindo Classification in reduced-port versus multi-port laparoscopic colectomy.

**Supplementary Figure 27:** Forest plot of postoperative complications classified as Grade 2 Clavien-dindo Classification in reduced-port versus multi-port laparoscopic colectomy.

**Supplementary Figure 28:** DOI plot of postoperative complications classified as Grade 2 Clavien-dindo Classification in reduced-port versus multi-port laparoscopic colectomy.

**Supplementary Figure 29:** Forest plot of postoperative complications classified as Grade 3 Clavien-dindo Classification in reduced-port versus multi-port laparoscopic colectomy.

**Supplementary Figure 30:** DOI plot of postoperative complications classified as Grade 3 Clavien-dindo Classification in reduced-port versus multi-port laparoscopic colectomy.

**Supplementary Figure 31:** Forest plot of postoperative complications classified as Grade 4 Clavien-dindo Classification in reduced-port versus multi-port laparoscopic colectomy.

**Supplementary Figure 32:** DOI plot of postoperative complications classified as Grade 4 Clavien-dindo Classification in reduced-port versus multi-port laparoscopic colectomy.
