# Supplementary material for: Reduced‐Port Versus Multi‐Port Laparoscopic Colectomy for Colorectal Cancer: A Systematic Review, Meta‐Analysis, and Meta‐Regression
Source: Ann Gastroenterol Surg. 2026 Aug 18;10(6):1712–21. doi: 10.1002/ags3.70264 (PMC13731019; doi:10.1002/ags3.70264)
Supplement: Supplementary file 2 [file AGS3-10-1712-s002.docx]

| **Author** | **Year** | **Country** | **Study design** | **Sample size** | | **Male, n(%)** | | **Female, n(%)** | | **Age, Mean (SD), Year** | | **BMI (kg/m2), Mean (SD)** | | **ASA score, n(%)** | | **History of Abdominal Operation, n (%)** | | **TNM stage, n(%)** | | **Tumor Size (cm)** | | **Tumor Location** | | | | **Number of ports in RPLC** | **Tumor Sites** | |
| --- | --- | --- | --- | --- | --- | --- | --- | --- | --- | --- | --- | --- | --- | --- | --- | --- | --- | --- | --- | --- | --- | --- | --- | --- | --- | --- | --- | --- |
|  |  |  |  | **RPLC** | **MPLC** | **RPLC** | **MPLC** | **RPLC** | **MPLC** | **RPLC** | **MPLC** | **RPLC** | **MPLC** | **RPLC** | **MPLC** | **RPLC** | **MPLC** | **RPLC** | **MPLC** | **RPLC** | **MPLC** | **RPLC** | | **MPLC** | |  | **RPLC** | **MPLC** |
|  |  |  |  |  |  |  |  |  |  |  |  |  |  |  |  |  |  |  |  |  |  | **Left** | **Right** | **Left** | **Right** |  |  |  |
| **Hasegawa et al.** | 2013 | Japan | Retrospective study | 28 | 62 | 18(64.3) | 40(64.5) | 10(35.7) | 22(35.5) | 68(44-81) | 70(42-85) | 23.0 ± 3.0 | 22.5 ± 3.4 | I:8(28.6), II:16(57.1), III:4(14.3), IV:0(0.0) | I:20(32.2), II:37(59.7), III:5(8.1), IV:0(0.0) | 8(28.6) | 20(32.3) | 0:3(10.7), I:8(28.6), II:11(39.3), III:6(21.4), IV:0(0.0) | 0:2(3.2), I:26(42.0), II:19(30.6), III:14(22.6), IV:1(1.6) | 3 | 2.8 | 0 | 15 | 1 | 17 | 3 | Ileocecal:3, Tansverse colon:0, Sigmoid colon:6, Anterior Resection:4 | Ileocecal:1, Tansverse colon:8, Sigmoid colon:27, Anterior Resection:8 |
| **Tashiro et al.** | 2015 | Japan | Retrospective study | 106 | 429 | 55(52) | 252(59) | 51(48) | 177(41) | 67.1 | 67.9 | 22.7 | 23.1 | - | - | 39(37) | 172(40) | 0/I:28 (26), II:28 (26), III:44 (42), IV:6 (6) | 0/I:122 (28), II:105 (25), III:147 (34), IV:55 (13) | 3.8(0.0) | 4.0(0.0) | 42 (40 ) | 46 (43) | 174 (41) | 144 (34 ) | 2 | Transverse colon:5 (5), Rectum:13 (12 ) | Transverse colon:18 (4), Rectum:93 (21 ) |
| **Liu et al.** | 2016 | China | Retrospective study | 22 | 25 | 12(54.5) | 13(52) | 10(45.5) | 12(48) | 64.2 ± 6.7 | 62.1 ± 9.8 | - | - | - | - | - | - | T1:1(4.5), T2:2(9.0), T3:7(31.8), T4:11(50.0) | T1:2(8), T2:3(12), T3:6(24), T4:14(56) | 3.5±0.8 | 3.7±0.9 | - | - | - | - | 3 | - | - |
| **Song et al.** | 2016 | Republic of Korea | Retrospective study | 32 | 217 | 19 (59.4) | 128 (59.0) | 13 (40.6) | 89 (41.0) | 65.2 ± 10.8 | 64.6 ± 12.7 | 23.3 ± 2.8 | 23.9 ± 3.9 | I:1 (3.1), II:29 (90.6), III:2 (6.3), IV:0(0.0) | I:8 (3.7), II:193 (88.9), III:16 (7.4), IV:0(0.0) | - | - | I:12 (37.5), II:7 (21.9), III:7 (21.9), IV:6 (18.7) | I:31 (14.3), II:71 (32.7), III:79 (36.4), IV:36 (16.6) | 3.6 ± 3.0 | 5.6 ± 3.0 | 22 (68.8) | 10 (31.2) | 147 (67.7) | 70 (32.3) | 2 | - | - |
| **Yu et al.** | 2016 | Republic of Korea | Retrospective study | 38 | 92 | 26 (68.4) | 45 (48.9 ) | 12 (31.6 ) | 47 (51.1) | 65.7±11.1 | 65.4±10.4 | 23.6±3.1 | 24.6±3.0 | I-II:33 (86.8), III:5 (13.2), IV:0(0.0) | I-II:76 (82.6), III:16 (17.4), IV:0(0.0) | 11 (28.9) | 31 (33.7) | I:12, II:25, III:13, IV:0 | I:32, II:56, III:36, IV:7 | 3.9±2.0 | 4.5±2.5 | 16 (42.1) | 22 (57.9) | 61 (66.3) | 31 (33.7) | 3 | - | - |
| **Oh et al.** | 2018 | Republic of Korea | Retrospective study | 115 | 490 | 61(53.0) | 308(62.9) | 54(47.0) | 182(37.1) | 61.0 ± 10.8 | 62.0 ± 10.9 | 24.3 ± 3.7 | 24.1 ± 3.1 | I-II:113(98.3), III:2(1.7), Iv:0(00.0) | I-II:476(97.1), III:14(2.9), IV:0(0.0) | 24(20.9) | 128(26.1) | I:46, II:59, III:56, IV:1 | I:161, II:276, III:213, IV:5 | 3.8 ± 2.1 | 4.2 ± 2.1 | - | - | - | - | 3 | - | - |
| **Kang et al. A** | 2018 | Republic of Korea | Retrospective study | 72 | 72 | 49(68.1) | 48(66.7) | 23(31.9) | 24(33.3) | 65.2 ± 9.9 | 66.1 ± 10.6 | 24.4 ± 3.0 | 24.3 ± 3.3 | I:20 (27.8), II:51 (70.8), III:1 (1.4), IV:0(0.00) | I:18 (25.0), II:54 (75.0), III:0 (0.0) IV:0(0.0) | 9 (12.5) | 10 (13.9) | I:29, II:41, III:31, IV:3 | I:22, II:43, III:29, IV:4 | 4.07 ± 2.13 | 4.31 ± 2.05 | - | - | - | - | 3 | Anterior resection for Rectal tumor | Anterior resection for Rectal tumor |
| **Kang et al. B** | 2018 | Republic of Korea | Retrospective study | 23 | 23 | 10 (43.5) | 11 (47.8) | 13 (56.5) | 12 (52.2) | 70.0 ± 8.0 | 71.9 ± 10.7 | 23.1 ± 3.4 | 23.5 ± 3.7 | I:6 (26.1), II:15 (65.2), III:2 (8.7), IV:0(0.0) | I:5 (21.7), II:17 (73.9), III:1 (4.3), IV:0(0.0) | 22 (95.7) | 22 (95.7) | I:6, II:14, III:9, IV:0 | I:12, II:15, III:8, IV:0 | 4.81 ± 2.16 | 4.99 ± 2.07 | - | - | - | - | 3 | Right Hemicolectomy for right colon tumor | Right Hemicolectomy for right colon tumor |
| **Kim et al.** | 2018 | Republic of Korea | Retrospective study | 74 | 89 | 38(51.3) | 43(48.3) | 36(48.7) | 46(51.7) | 69.0 ± 11.5 | 69.4 ± 8.6 | 22.6 ± 3.0 | 23.9 ± 3.2 | I:0(0.0), II:54 (73.0), III:20 (27.0), IV:0(0.0) | I:0(0.0), II:66 (74.2), III:22 (24.7), IV:1 (1.1) | 13(17.6) | 20(22.5) | I:9 (12.2), II:29 (39.2), III:24 (32.4), IV:12 (16.2) | I:35 (39.3), II:33 (37.1), III:16 (18.0), IV:5 (5.6) | 5.6±2.8 | 4.6±3.1 | - | - | - | - | 3 | Cecus:14 (18.9) , Ascending colon:39 (52.7) , Hepatic flexure:13 (17.6) , Transverse colon:8 (10.8) | Cecus:14 (15.7) , Ascending colon:43 (48.3) , Hepatic flexure:15 (16.9) , Transverse colon:17 (19.1) |
| **Shi et al.** | 2019 | China | Retrospective study | 39 | 39 | 16(41.1) | 18(46.2) | 23(58.9) | 21(53.8) | 65.54 ± 8.65 | 66.54 ± 9.50 | 23.10 ± 2.81 | 22.87 ± 3.32 | I-II:32 (82.1), III-IV:7 (17.9) | I-II:31 (79.5), III-IV:8 (20.5) | 13(33.3) | 12(30.8) | I-II:22 (56.4), III-IV:17 (43.6) | I-II:24 (61.5), III-IV:15 (38.5) | 4.47 ± 1.69 | 4.27 ± 1.60 | - | - | - | - | 3 | - | - |
| **Zhang et al.** | 2021 | China | Retrospective study | 86 | 96 | 41(47.7) | 46(47.9) | 45(52.3) | 50(52.1) | 62(17) | 66(13) | <18.50 (%):7 (8.1) ,18.50–24.99 (%):61 (70.9) ,>24.99 (%):18 (20.9) | <18.50 (%):3 (3.1) ,18.50–24.99 (%):60 (62.5) ,>24.99 (%):33 (34.4) | I-II:69 (80.2), III-IV:17 (19.8) | I, II:80 (83.3), III, IV:16 (16.7) | 25(29.1) | 33(34.4) | I:3 (3.4), II:50 (7.5), III:33 (37.9) | I:11 (11.3), II:52 (53.6), III:33 (34.0) | 4.0 (2.0) | 4.5 (2.0) | - | - | - | - | 3 | Ileocecus:21 (24.4), Ascending colon:33 (38.4), Hepatic flexure:24 (27.9), Transverse colon:8 (9.3) | Ileocecus:34 (35.4), Ascending colon:21 (21.9), Hepatic flexure:33 (34.4), Transverse colon:8 (8.3) |
| **Wu et al.** | 2024 | China | Retrospective study | 63 | 52 | 35 (55.56) | 27 (51.92) | 28 (44.44) | 25 (48.08) | 55.02 ± 10.97 | 54.10 ± 9.56 | 24.05 ± 3.04 | 23.73 ± 2.58 | - | - | - | - | I:0(0.0), II:20 (31.75), III:27 (42.86), IV:16 (25.40) | I:0(0.0), II:19 (36.54), III:18 (34.62), IV:15 (28.85) | - | - | 15 (23.81) | 31 (49.21) | 18 (34.62) | 20 (38.46) | 2 | Rectum:17 (26.98) | Rectum:14 (26.92) |
| **Fujii et al.** | 2025 | Japan | Randomized controlled trial | 45 | 46 | 26 (57.8) | 25 (54.3) | 19(42.2) | 21(45.7) | 75 (35–94) | 69 (25–87) | 24.2(16.5–32.0) | 23.9(16.2–38.2) | I:12, II:31, III:2, IV:0 | I:15, II:30, III:1, IV:0 | - | - | 0:1(2.2), I:19(42.2), II:10(22.2), III:15(33.3), IV:0(0.0) | 0:1(2.2), I:17 (37.0), II:10 (21.7), III:18 (39.1), IV:0(0.0) | 3.1 (0.5–13.0) | 2.9 (1.0–8.0) | 28 (62.2) | 17 (37.8) | 31 (67.4) | 15 (32.6) | 2 | - | - |
| **Liu et al.** | 2025 | China | Retrospective study | 211 | 211 | 124(58.8) | 123(58.3) | 87(41.2) | 88(41.7) | 59.7 ± 12.4 | 59.0 ± 12.8 | 22.8 ± 3.1 | 22.8 ± 3.0 | I:6 (2.8), II:184 (87.2), III:20 (9.5), IV:1 (0.5) | I:11 (5.2), II:180 (85.3), III:17 (8.1), IV:3 (1.4) | - | - | I:15 (7.1), II:64 (30.3), III:93 (44.1), IV:39 (18.5) | I:21 (10.0), II:65 (30.8), III:87 (41.2), IV:38 (18.0) | - | - | 89 (42.2) | 53 (25.1) | 80 (37.9) | 61 (28.9) | 3 | Rectum:69(31.4) | Rectum:70(32.5) |

**Table 1:** Baseline characteristics of the included studies.

RPLC: Reduced-Port Laparoscopic Colectomy; MPLC: Multi-Port Laparoscopic Colectomy; BMI: Body Mass Index; kg/m²: Kilograms per square meter; ASA: American Society of Anesthesiologists Physical Status Classification; TNM: Tumor, Node, Metastasis staging system; I–IV: Stages in ASA or TNM classification

*****Values are presented as mean ± standard deviation (SD) or median (range), as reported in individual studies.
